# Plant Responses and Tolerance to Salt Stress: Physiological and Molecular Interventions 2.0

**DOI:** 10.3390/ijms242115740

**Published:** 2023-10-30

**Authors:** Mirza Hasanuzzaman, Masayuki Fujita

**Affiliations:** 1 Department of Agronomy, Faculty of Agriculture, Sher-e-Bangla Agricultural University, Dhaka 1207, Bangladesh; 2 Laboratory of Plant Stress Responses, Department of Plant Science, Faculty of Agriculture, Kagawa University, Takamatsu 761-0795, Kagawa, Japan

Environmental problems are pervasive and significantly impact a variety of plant species, which are affected by two broad types of conditions: abiotic and biotic stress. Abiotic stresses commonly arise due to climatic shifts and have detrimental impacts on plant growth and metabolic processes [1]. These stresses include but are not limited to water scarcity, variations in temperature, radiation, flooding, heavy metals, and changes in soil pH and salinity.

One of the most critical forms of environmental stress is salt stress, which is particularly prevalent in coastal areas. It has emerged as a significant environmental issue due to rising levels of soil salinity. This stress condition has adversely affected 33% of irrigated lands and 20% of all cultivated lands around the globe [2]. When the condition is severe, it can halt crop production entirely, reduce yields by 10 to 25%, and can even lead to desertification [3]. The causes of salt stress are manifold, including poor drainage systems and insufficient rainfall, among others. Intensive irrigation practices result in secondary salinization and alkalinization of soils, aggravating the issue further. As the salt concentration increases, crop yields decline due to the inability to leach salt out of the soil. Other contributing factors include inherent soil salinity, seawater intrusion, surface evaporation, plant transpiration, and excessive fertilizer use. Soluble salts such as sodium, calcium, and magnesium chlorides and sulfates are typically found in saline soils, while alkali soils contain sodium silicates, carbonates, and bicarbonates. Salt stress impacts critical metabolic processes including photosynthesis, protein synthesis, and lipid metabolism [4]. It not only increases the susceptibility of plants to diseases but also leads to ion toxicity, nutritional imbalances, and oxidative stress [5].

Plant species exhibit different tolerances to salt stress. Glycophytes cannot thrive in high salt concentrations, whereas halophytes are more salt-tolerant [6]. Halophytes are further classified into extreme euhalophytes and intermediate oligohalophytes based on their varying levels of resistance. Physiological traits related to adaptation in high salt conditions among halophytes include internal salt concentrations, the control of turgor pressure through organic solute synthesis, and sometimes, the development of salt-excreting glands. A significant number of crop species are sensitive to salinity, and their responses vary considerably [7]. Even NaCl concentrations much lower than seawater can severely impede the growth of many crops [8]. Salinity affects plants through various mechanisms, including osmotic inhibition, direct toxicity, and nutritional impacts, among others. Some plants can defend against salt stress through specific strategies such as ion homeostasis and compartmentalization [9]. In a nutshell, salinity is an increasing concern for global agriculture with no straightforward solution. Ongoing research and technological advancements are crucial for mitigating this complex issue effectively.

This Special Issue of the *International Journal of Molecular Sciences*, entitled “Plant Responses and Tolerance to Salt Stress: Physiological and Molecular Interventions 2.0”, features a total of five contributions. These include three original articles [10,11,12] and two reviews [13,14], which offer fresh insights into the latest advancements in salt stress responses and tolerance, with a special emphasis on molecular physiology.

Wang et al. [10] aimed to understand the molecular and metabolic processes regulating salt tolerance in ZM-4, a salt-tolerant resource. The researchers analyzed the transcriptome and metabolome of ZM-4’s roots and leaves under salt stress. They observed an increase in fresh weight following NaCl treatment and increased antioxidant capacity in the leaves. ZM-4 also exhibited upregulated genes linked to flavonoid synthesis pathways and higher flavonoid content. In the roots, higher expression of genes related to amino acid and sugar pathways facilitated excellent osmotic adjustment. These findings contribute to a deeper understanding of salt-tolerance pathways in ZM-4 and offer significant insights for breeding salt-tolerant rootstocks.

Mondal et al. [11] focused on the challenge of salinity stress in coastal rice cultivation, particularly when seawater intrudes inland during dry periods. They examined a mapping population derived from a cross between a salt-tolerant Saudi Arabian variety, Hasawi, and a salt-sensitive Bangladeshi variety, BRRI dhan28. Through genotyping by skim sequencing, 40 QTLs were identified, including three yield-related QTLs on chromosome 3 not previously associated with grain yield under saline conditions. The study aims to identify QTLs linked to salt tolerance in rice and provide valuable genetic resources for further research.

Ouertani et al. [12] explored the potential of barley landraces (Hordeum vulgare) as a source of tolerance genes. They compared the morpho-physiological traits of salt-tolerant (Boulifa) and salt-sensitive (Testour) genotypes under salt stress conditions. Boulifa exhibited better antioxidant protection and higher antioxidant enzyme activities against salt stress. Transcriptome analysis revealed differential expression patterns between the two genotypes, with several salt-responsive genes being induced only in Boulifa but suppressed in Testour. The study concluded that these differential transcriptomic responses are crucial for breeding salt-tolerant barley varieties.

Dai et al. [13] reviewed advancements in omics studies related to salt tolerance in rice. The review emphasizes the importance of understanding the molecular mechanisms behind rice’s salt tolerance and discusses how integrating multi-omics data can advance our understanding of rice’s defense mechanisms against salt stress. The paper suggests that future research should focus on incorporating innovative approaches like microbiology, phenomics, and artificial intelligence to address current challenges.

Gupta et al. [14] examined how plants, during their life cycle, are exposed to various abiotic stressors such as drought and salinity, which can adversely affect their survival. The review highlights the capacity of plant growth-promoting bacteria (PGPB) to mitigate the impacts of these stressors. PGPB inoculation has been shown to enhance plant development through various mechanisms and to counteract stress-induced oxidative damage. The study concludes that PGPB offers a promising avenue for addressing the challenges posed by climate change and for promoting sustainable agriculture.

Overall, the articles included in this Special Issue explore multiple dimensions of salt stress responses in plants. In addition, they delve into various mechanisms and strategies for enhancing plant salt tolerance. Such research offers new avenues for developing crop varieties suited to saline environments, particularly in the context of climate change.
